# Paranasal Volume Enhancement With Sticky Bone to Improve Nasolabial Fold Aesthetics in Orthognathic Surgery Patients: A Case Report

**DOI:** 10.1155/crid/2437983

**Published:** 2026-10-08

**Authors:** Hugo Leonardo Mendes Barros, Isabella Mousinho Marinho dos Santos, Ana Luisa Rios Barbosa de Almeida Xavier, Adelmar Pereira da Silva Neto, Thais Pimental de Sá Bahia, Daniel Amaral Alves Marliére

**Affiliations:** ^1^ Oral and Maxillofacial Surgery Service Hospital São Marcos Teresina Piauí Brazil; ^2^ Department of Oral and Maxillofacial Surgery Rio de Janeiro State University Rio de Janeiro Rio de Janeiro Brazil uerj.br; ^3^ Department of Clinical Dentistry School of Dentistry Federal University of Juiz de Fora Juiz de Fora Minas Gerais Brazil ufjf.br

**Keywords:** bone graft, Le Fort osteotomy, nasolabial fold, orthognathic surgery, platelet-rich fibrin

## Abstract

**Purpose:**

The paranasal region is a frequent source of aesthetic complaints in patients with dentofacial deformities, and soft tissue projection in this area does not always follow the skeletal movements achieved in orthognathic surgery. Sticky Bone—a particulate bone graft matrix agglutinated by platelet‐rich fibrin (PRF)—has an established role in regenerative procedures in implant dentistry; however, to the best of the authors′ knowledge, its use for paranasal volume enhancement during orthognathic surgery had not been previously described. The present report introduces this novel application and documents its clinical and tomographic behavior.

**Case Reports:**

Two female patients, one with a Class II malocclusion and mandibular retrognathism (Case A) and the other with a Class III malocclusion, mandibular prognathism, and anteroposterior maxillary deficiency (Case B), presented with aesthetic complaints involving the paranasal region. The cases were assessed by means of intraoral and extraoral photographs, computed tomography, and occlusal scanning. Virtual surgical planning was performed with Dolphin Imaging software. During surgery, mandibular osteotomies were performed using the bilateral sagittal split technique, and a Le Fort I osteotomy was performed in the maxilla. After fixation of the osteotomized segments, a mixture of particulate bovine graft and PRF (Sticky Bone) was positioned over the canine pillars (lateral to the piriform aperture), between PRF and collagen membranes. Aesthetic outcomes were favorable, as indicated by the patients′ subjective satisfaction. On tomographic superimposition, graft thickness remained stable throughout follow‐up, with a maximum variation of 0.8 mm in Case A (18 months) and 0.6 mm in Case B (24 months), and no measurable change between the first and second years in Case B.

**Conclusion:**

Sticky Bone proved to be a feasible and effective technique for augmenting the paranasal region, contributing to aesthetic refinement. Its biocompatibility, stability, and low cost make it an excellent option for use as a bone graft between osteotomized bone segments.

## 1. Introduction

The three‐dimensional position of the maxilla directly influences facial harmony. Deficient paranasal support manifests clinically as bilateral deepening of the nasolabial fold and a flattened or concave profile [1], findings that are common in patients with anteroposterior maxillary deficiency and that convey an aged appearance. Orthognathic surgical planning must, therefore, take into account that the volume of the paranasal region is decisive for achieving a balanced and youthful facial contour.

The Le Fort I osteotomy is the standard procedure for repositioning the maxilla. Technical modifications intended to increase paranasal projection have been described, including the quadrangular osteotomy [2], elevation of the osteotomy level [3], the U‐shaped osteotomy [4], and the pyriform rim sandwich osteotomy [5]. Even so, the soft tissue projection obtained in the paranasal area does not always follow the displacement of the osteotomized segments, which may leave the patient′s aesthetic expectations unmet [6, 7].

In such situations, additional refinement may be pursued with alloplastic onlay materials such as polymethylmethacrylate and porous polyethylene [8–10], temporary injectable fillers based on hyaluronic acid [11–13] or calcium hydroxylapatite [14], autologous fat transfer [15], and autogenous bone blocks fixed to the anterior maxillary surface [16, 17]. Each of these alternatives, however, carries recognized drawbacks: Alloplastic implants are associated with infection and foreign body reactions [18], injectable fillers offer limited clinical durability [12, 19], block grafts require a donor site and osteosynthesis fixation [16, 17], and fat grafting behaves unpredictably because of short‐ and medium‐term resorption [15].

In recent years, several dental specialties have adopted platelet‐rich fibrin (PRF) to enhance the regeneration of bone and soft tissues. When PRF‐derived fibrin is combined with a granular bone substitute, the resulting agglutinated mass—known as Sticky Bone—has shown favorable clinical performance in alveolar ridge preservation, horizontal and vertical ridge augmentation, and maxillary sinus floor elevation for implant rehabilitation [20–22]. Sticky Bone constitutes a moldable graft matrix in which growth factors remain entrapped within the fibrin network, combining biocompatibility, volumetric durability, and low cost [23, 24].

The purpose of this report is to present the use of Sticky Bone as a definitive adjunct for augmentation of the paranasal region in patients undergoing orthognathic surgery—an application that, to the best of the authors′ knowledge, had not been described in the literature—detailing the technique in a reproducible manner and documenting its clinical and tomographic behavior, with a view to greater aesthetic refinement of the paranasal region and, simultaneously, to supporting healing and consolidation between the osteotomized bone segments.

## 2. Case Report

Two patients presented to the Oral and Maxillofacial Surgery and Traumatology Clinic of Hospital São Marcos, Teresina, Piauí, Brazil, with dentofacial deformities, aesthetic complaints, and functional limitations. Neither patient had a significant medical history, and no comorbidities or contraindications to surgery were identified. Facial and dental analysis was performed in centric relation with the lips at rest, following the parameters described by Arnett and Bergman [25], and the clinical characteristics of the facial tissues were recorded (Table 1). Both patients signed a free and informed consent form covering the treatment and the use of their images for scientific publication, and all procedures complied with the ethical principles of the Declaration of Helsinki of the World Medical Association.

**Table 1:** Clinical characteristics of the patients recorded during facial analysis.

Facial reference	Quantitative and qualitative data of the facial analysis	
	Case A	Case B
Upper incisor exposure at rest	4.5 mm	2.1 mm
Dental and gingival exposure in smile	10.5 mm	7.8 mm
Maxillary midline	Coincident with the center of the chin	Deviation to the right relative to the center of the chin
Mandibular midline	Coincident with the center of the chin	Deviation to the right relative to the center of the chin
Symphysis height	45 mm	46 mm
Height of the middle third of the face	48 mm	44 mm
Height of the lower third of the face	72 mm	67 mm
Upper lip support	Normal	Deficient
Nasolabial angle	Greater than 90°	Greater than 90°
Paranasal region	Deficient	Deficient
Open bite	Absent	Absent
Overjet	3.2 mm	−2.5 mm
Overbite	2.8 mm	−1.2 mm
Spee curve	Normal	Normal

### 2.1. Case A

A 37‐year‐old female patient complained of a “small chin, difficulty sealing the lips passively, teeth that do not fit together, and poor breathing and sleep.” Clinical and imaging assessment revealed an Angle Class II skeletal pattern with mandibular retrognathism, anteroposterior maxillary excess, Class II malocclusion, and an obtuse cervicomental angle (Figure 1). Despite the maxillary protrusion, the patient presented a deep nasolabial fold attributable to her maxillary anatomy, which combined anteroposterior excess of the alveolar segment with deficiency in the canine fossa region. Her clinical features are summarized in Table 1.

**Figure 1:**
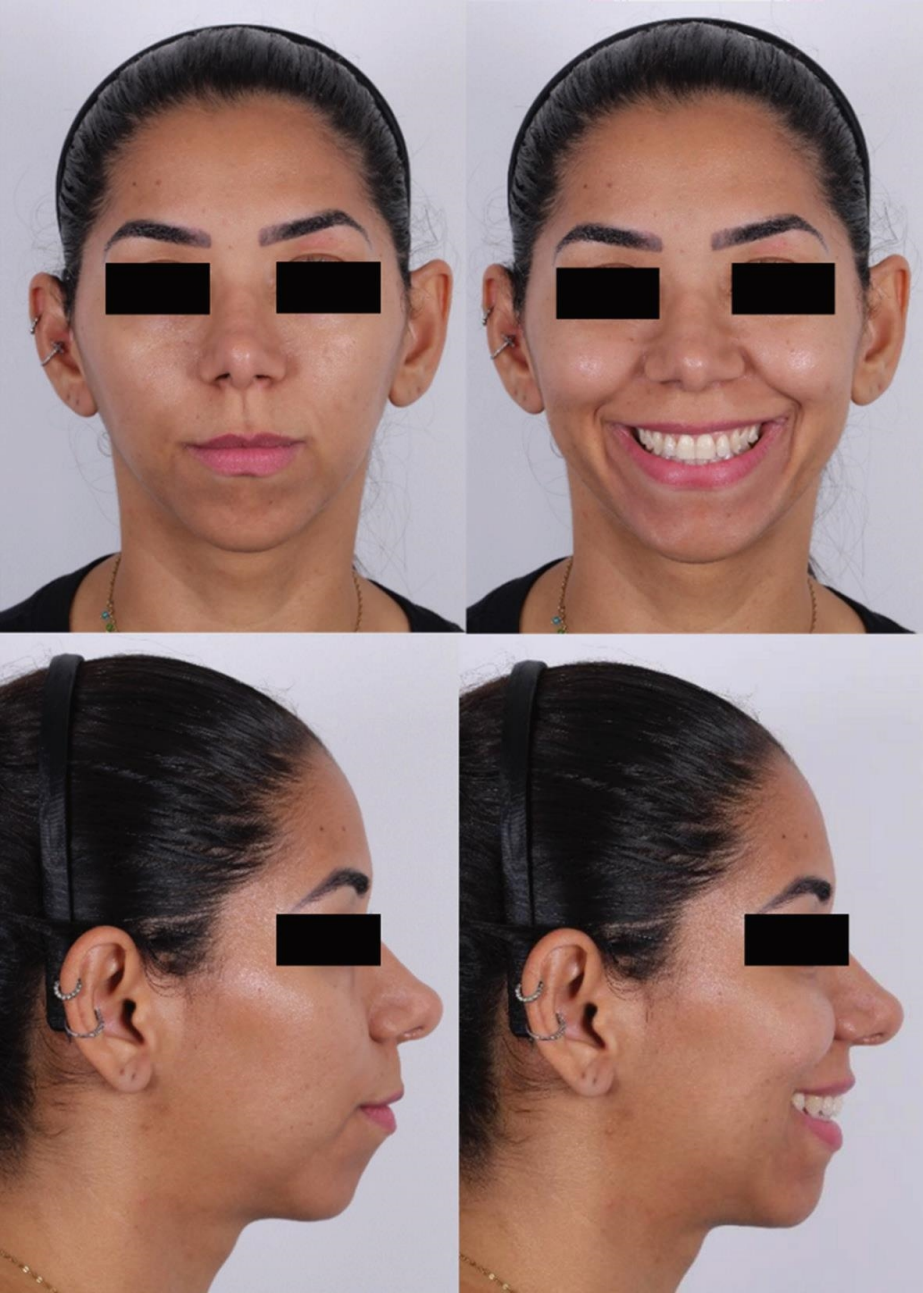
Preoperative extraoral photographs—Case A.

### 2.2. Case B

A 36‐year‐old female patient complained of a “large jaw, a deep nasolabial fold with an aged appearance, and teeth that no longer fit together after previous orthodontic treatment.” She presented an Angle Class III skeletal pattern with mandibular prognathism, anteroposterior maxillary deficiency, and Class III malocclusion (Figure 2). Her clinical features are likewise summarized in Table 1.

**Figure 2:**
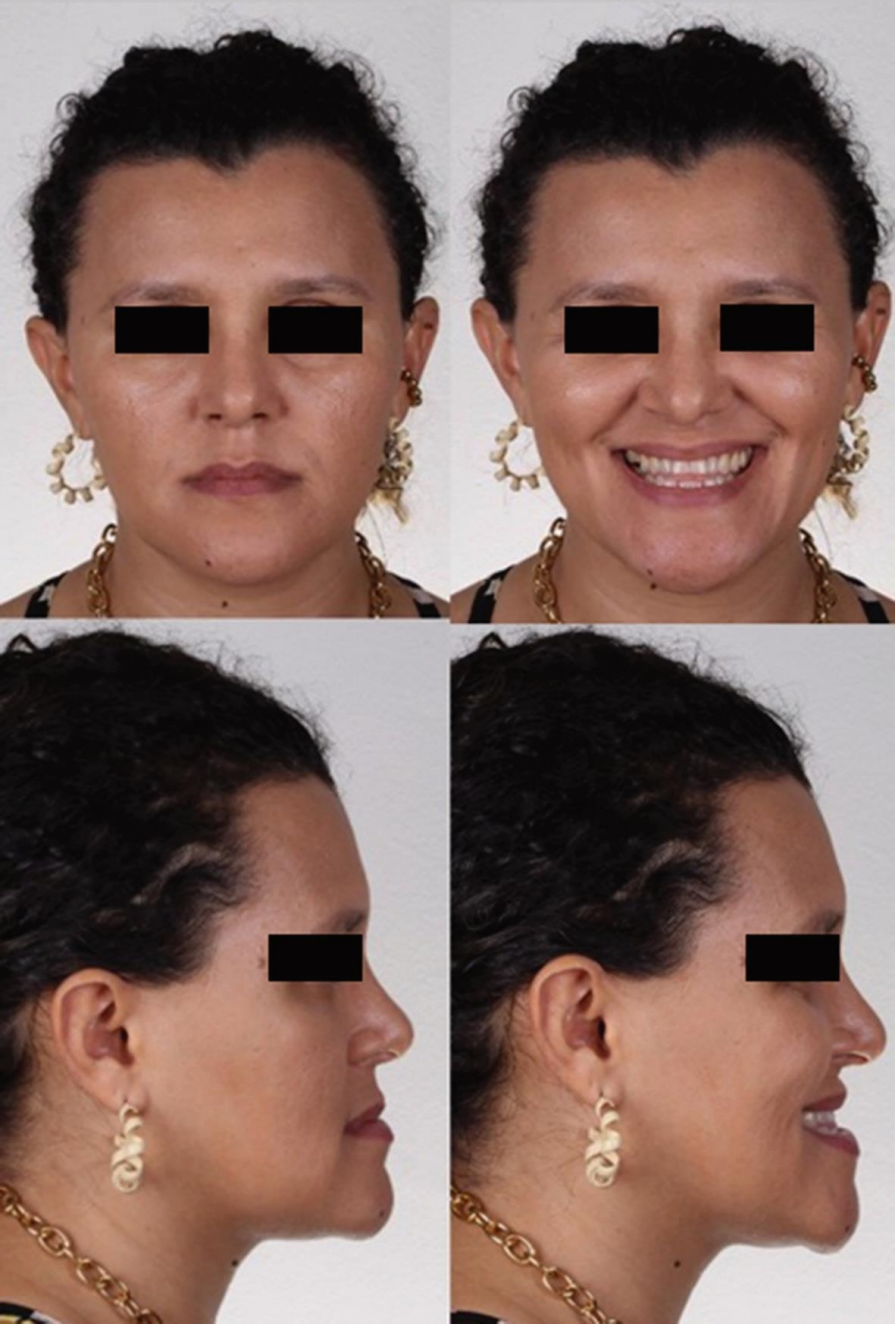
Preoperative extraoral photographs—Case B.

### 2.3. Virtual Planning

Both patients underwent image acquisition with a multidetector helical computed tomography scanner (Siemens Emotion, 16 channels; Siemens Healthcare Diagnósticos Ltda., Brazil). The DICOM (Digital Imaging and Communications in Medicine) files were reconstructed into three‐dimensional models in Dolphin Imaging 3D software, Version 11.7 (Dolphin Imaging and Management Solutions, Chatsworth, California, United States), and digital impressions of the dental arches were obtained with a 3Shape TRIOS intraoral scanner.

### 2.4. Case A

A counterclockwise rotation of the maxillomandibular complex was planned with a mandible‐first sequence: bilateral sagittal split osteotomy of the mandibular ramus, fabrication of the intermediate occlusal guide, Le Fort I osteotomy of the maxilla, and fabrication of the final guide (Figure 3). The plan established a mandibular advancement of +13.10 mm at pogonion, an advancement genioplasty of +7.08 mm, and a maxillary impaction of −1.00 mm (Table 2).

**Figure 3:**
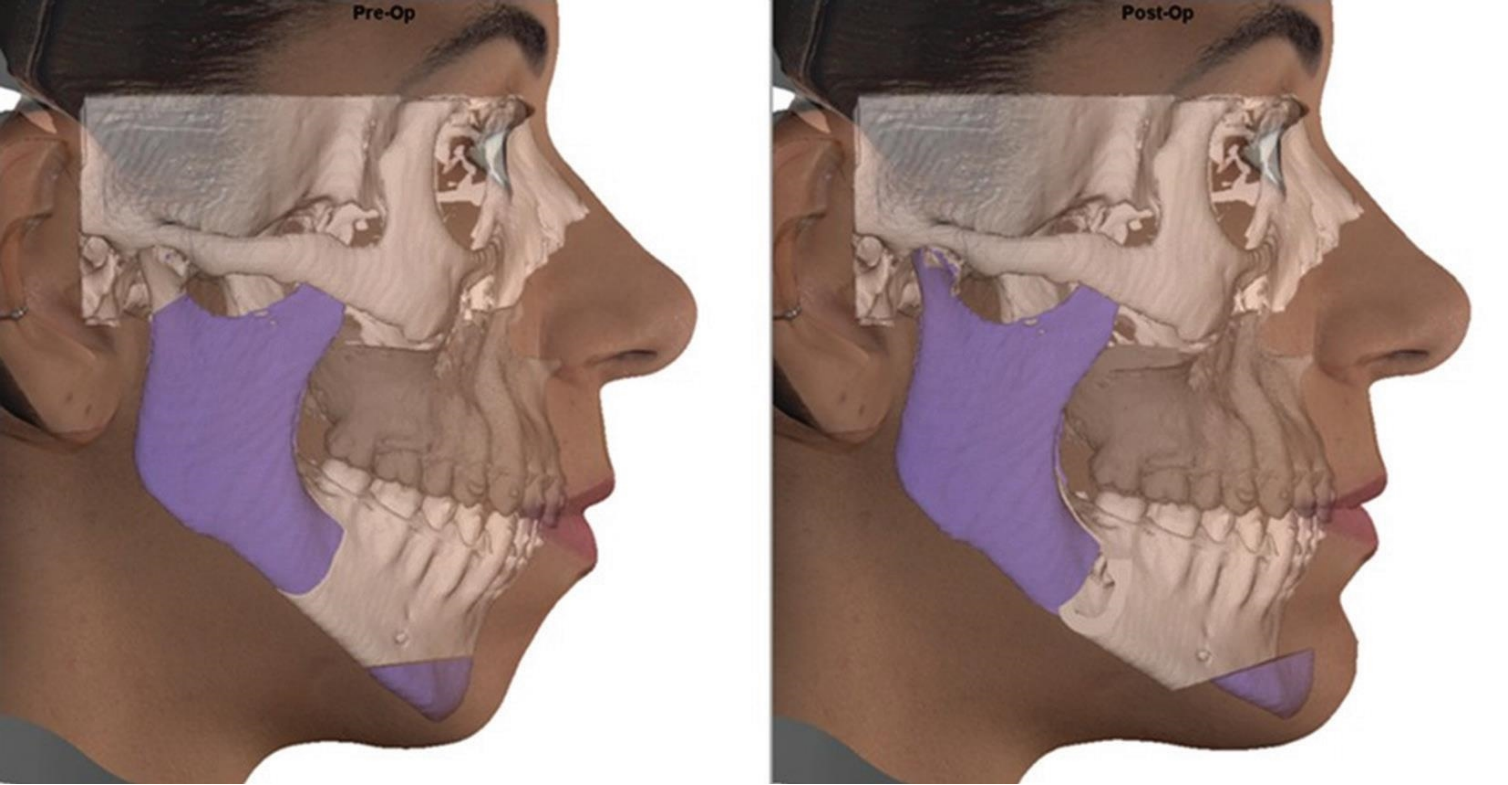
Virtual surgical planning images—Case A.

**Table 2:** Linear measurements of the virtual surgical planning movements, in millimeters (mm).

Cephalometric references	Case A			Case B		
	A+/P−	L+/R−	D+/U−	A+/P−	L+/R−	D+/U−
ANS	−4.23	−0.56	−1.00	+6.97	+0.19	+2.31
PNS	−3.87	−0.46	+5.38	+5.58	−1.30	−1.64
Upper incisor tip midpoint	0	−0.02	−1.00	+3.41	+1.00	+2.64
A point	−2.73	−0.37	−0.79	+5.79	+0.27	+2.10
B point	+4.57	+1.03	−0.18	−0.32	+0.16	+0.58
Pogonion	+13.10	+1.23	+1.84	−1.26	+0.36	+2.94
Lower incisor tip midpoint	+0.80	+0.70	+1.42	−0.03	−0.18	+0.58
Genioplasty	+7.08	+0.04	+2.27	−0.79	0	+2.36

Abbreviations: A, anterior; D, down; L, left; P, posterior; R, right; U, up.

### 2.5. Case B

A mandibular setback with clockwise rotation of the maxillomandibular complex was planned, also with a mandible‐first sequence: bilateral sagittal split osteotomy of the mandibular ramus, intermediate occlusal guide, three‐segment Le Fort I osteotomy, and final guide (Figure 4). The plan established a mandibular setback of −1.26 mm at pogonion, a maxillary advancement of +3.41 mm, and a vertical increase of +2.64 mm (Table 2).

**Figure 4:**
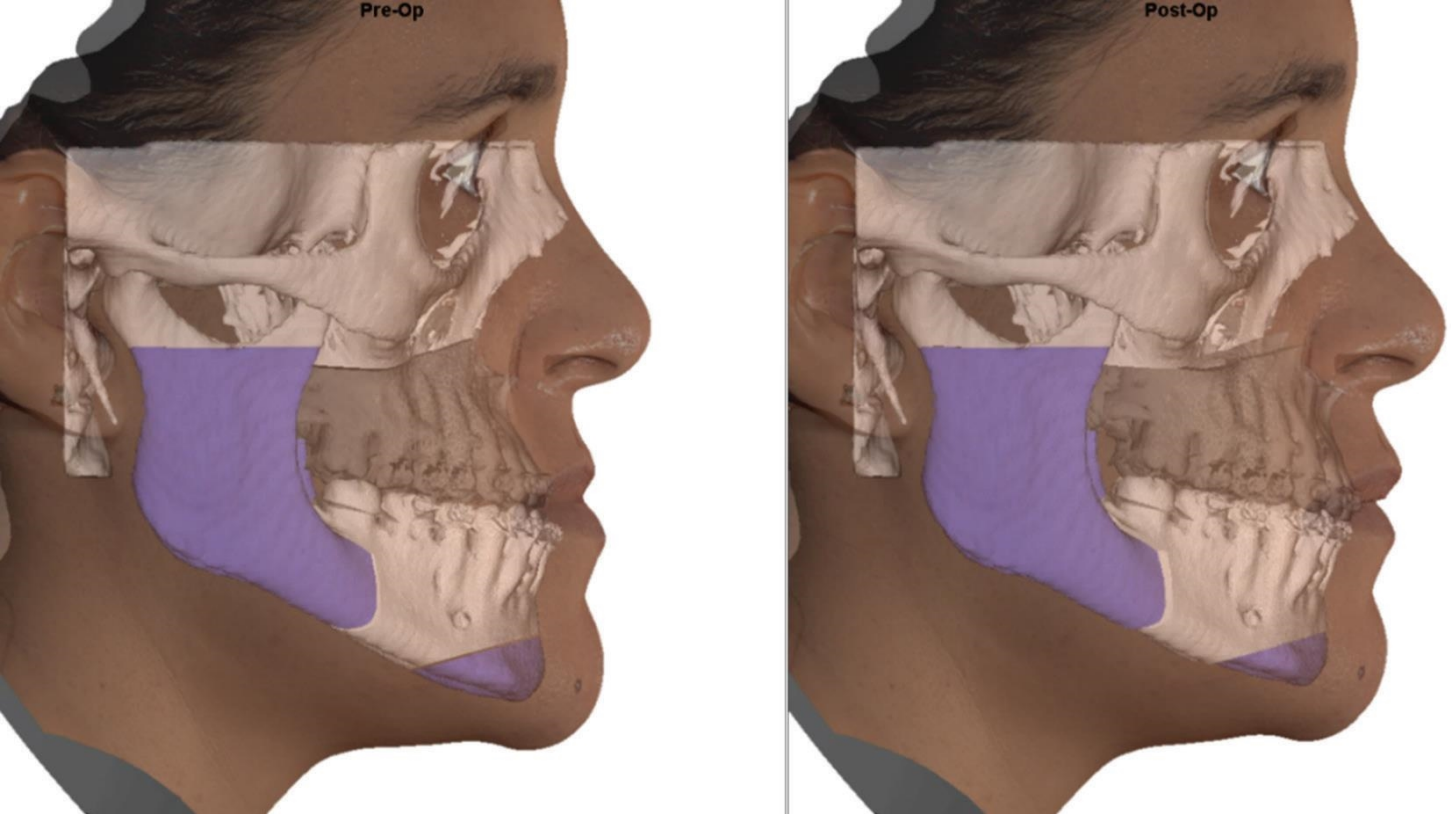
Virtual surgical planning images—Case B.

### 2.6. Surgical Procedure

Both operations were performed in a hospital setting under general anesthesia by the same surgical team, following the planned mandible‐first sequence and reproducing the virtual plan by means of the interocclusal guides positioned intraoperatively. In Case A, each osteotomized mandibular ramus was stabilized with one straight plate fixed with four monocortical screws and two bicortical screws; in Case B, two straight plates were applied bilaterally with eight monocortical and two bicortical screws. In the maxilla, two L‐shaped plates and two straight plates fixed with 19 screws were used in Case A, whereas Case B received four L‐shaped plates with fifteen 2.0‐mm screws and one additional plate with eight 1.5‐mm screws at the interdental osteotomy region. Rigid internal fixation employed a 2.0‐mm osteosynthesis system (Medartis AG, Basel, Switzerland).

During the same operative session, peripheral venous blood was collected and distributed into six 10‐mL silica‐coated tubes without anticoagulant (Process for PRF, by Choukroun; Implantec, Brazil), centrifuged in a PRF Duo Quattro centrifuge (Implantec, Brazil) at 1300 rpm for 14 min, and into two white‐cap plastic vacuum tubes (Process for PRF, by Choukroun; Implantec, Brazil), centrifuged at 700 rpm for 5 min, in accordance with the low‐speed centrifugation concept [26].

After centrifugation of the silica‐coated tubes, the L‐PRF clot was gently separated from the red cell fraction with tweezers and scissors (Figure 5a). The clots were placed on the tray of the PRF Box kit (PRF Choukroun protocol; Implantec, Brazil) and compressed with the stainless steel compactor, yielding fibrin membranes ready for use (Figure 5b).

**Figure 5:**
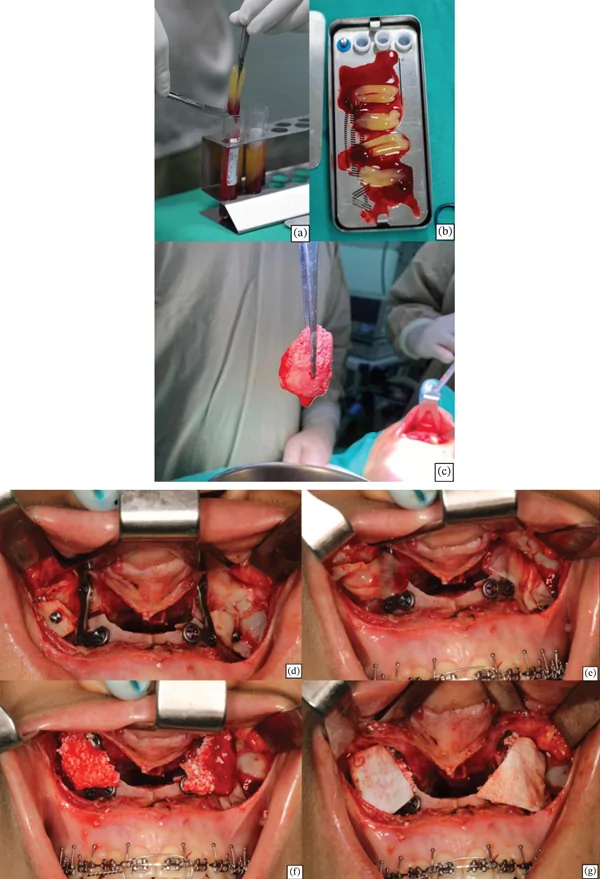
(a,b) L‐PRF membranes obtained after centrifugation and compression. (c) Sticky Bone produced by combining particulate bovine bone substitute with liquid fibrin. (d) Paranasal region after repositioning and fixation of the Le Fort I segments. (e) L‐PRF membranes positioned over the canine pillar, lateral to the piriform aperture. (f) Sticky Bone packed over the L‐PRF membranes. (g) Collagen membrane positioned over the Sticky Bone to support guided bone regeneration.

From the white‐cap tubes, liquid fibrin was aspirated with a pipette and added to 2 g of particulate deproteinized bovine bone mineral (Geistlich Bio‐Oss) on the Teflon dish of the PRF Box kit. The protein‐rich exudate released during membrane compression, containing vitronectin and fibronectin, was added to the mixture; this step promotes agglutination of the granules within a resistant fibrin network, producing a growth factor–enriched graft matrix—Sticky Bone—capable of enhancing bone and soft tissue regeneration (Figure 5c) [24].

Over the fixed Le Fort I segments, lateral to the piriform aperture (Figure 5d), the L‐PRF membranes were first adapted to seal the region and prevent displacement of graft particles toward the maxillary sinus (Figure 5e). Sticky Bone was then packed bilaterally over the canine pillars, on top of the L‐PRF membranes (Figure 5f). The amount inserted was estimated clinically by visual assessment and, therefore, differed between the two patients according to the magnitude of the paranasal deficiency identified in the facial analysis and to the surgeon′s judgment. Finally, 25 × 25 mm resorbable collagen membranes (Geistlich Bio‐Gide) were positioned bilaterally over the graft to support guided bone regeneration (Figure 5g).

### 2.7. Postoperative Period

The postoperative course was uneventful in both patients: There was no infection, wound dehiscence, or exposure of the grafted material, and no adverse or unanticipated events were recorded. Computed tomography obtained within the first 24 h after surgery confirmed adequate positioning of the maxillomandibular segments and osteosynthesis devices, as well as stable adaptation of the biomaterial in the paranasal region (Figure 6). In Case A, control tomographic scans were obtained at 6 and 18 months; in Case B, they were obtained at 12 and 24 months. The tomographic volumes were superimposed in Dolphin Imaging software according to the methods described by Marliére et al. [27] and Haas Júnior et al. [28], and graft thickness was measured bilaterally on axial sections at the level of the canine pillar (Figures 7 and 8).

**Figure 6:**
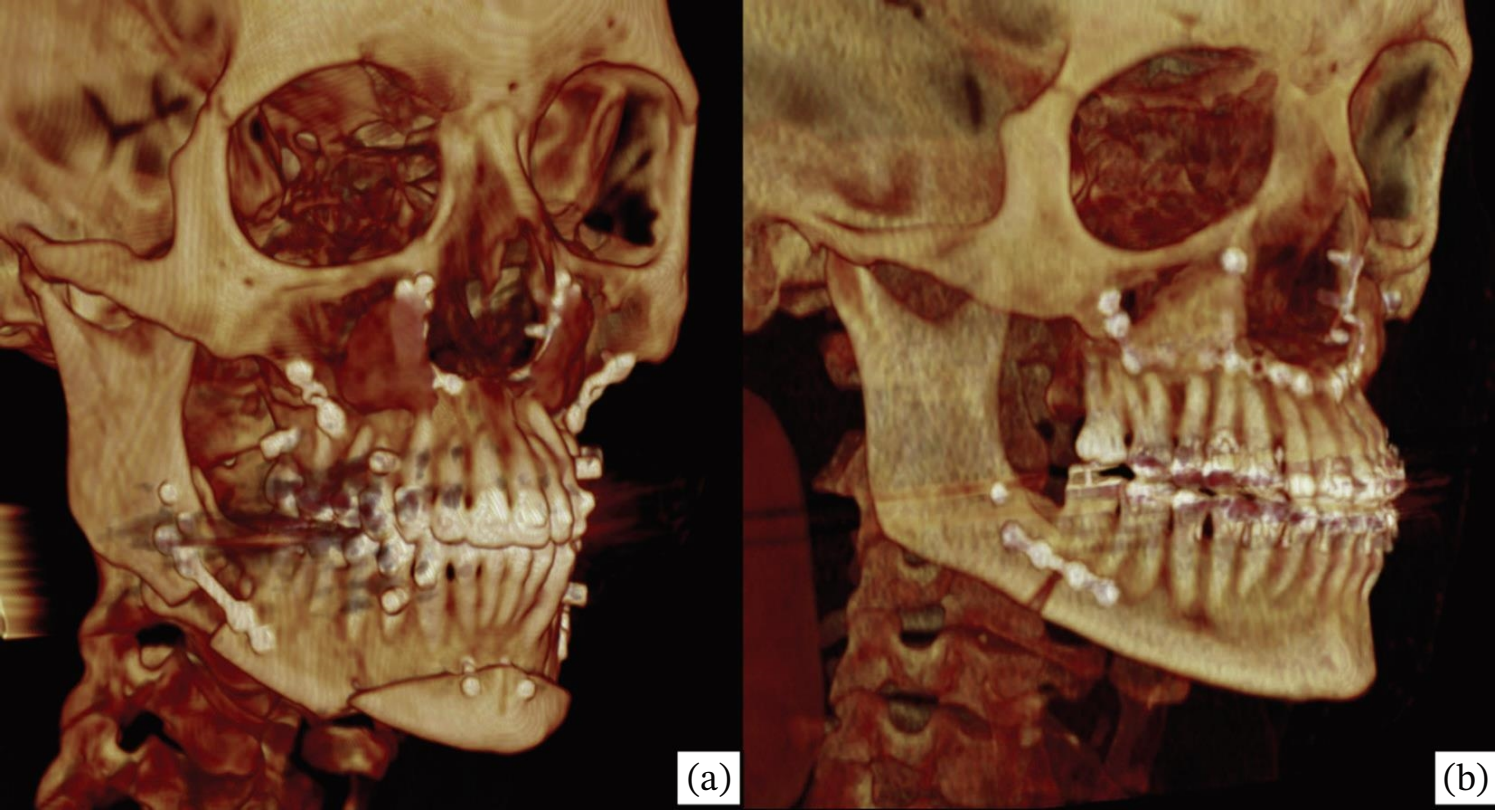
Three‐dimensional reconstructions of the immediate postoperative computed tomography scans: (a) Case A and (b) Case B.

**Figure 7:**
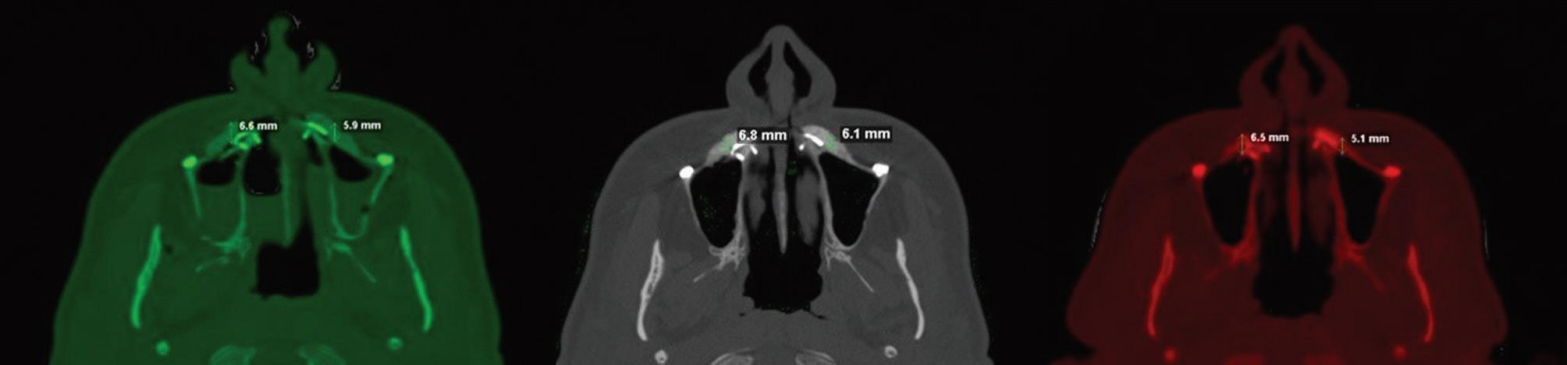
Superimposition of the computed tomography scans of Case A on axial sections at the level of the canine pillar: immediate postoperative period (green), 6 months (gray), and 18 months (red), with graft thickness measured bilaterally.

**Figure 8:**
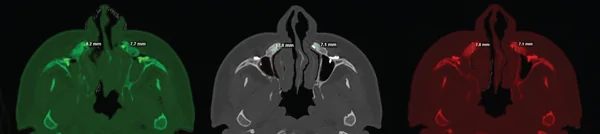
Superimposition of the computed tomography scans of Case B on axial sections at the level of the canine pillar: immediate postoperative period (green), 12 months (gray), and 24 months (red), with graft thickness measured bilaterally.

In Case A, graft thickness was 6.6 mm on the right and 5.9 mm on the left in the immediate postoperative period, 6.8 and 6.1 mm at 6 months, and 6.5 and 5.1 mm at 18 months, corresponding to a net variation of −0.1 mm on the right and −0.8 mm on the left relative to the immediate scan (Figure 7). In Case B, the values were 8.2 mm on the right and 7.7 mm on the left in the immediate postoperative period and 7.8 and 7.1 mm at 12 months, remaining identical at 24 months—that is, a variation of −0.4 mm on the right and −0.6 mm on the left, entirely occurring within the first year, with no measurable change between the first and second years of follow‐up (Figure 8).

From the aesthetic and functional standpoints, the outcomes were favorable: The depth of the nasolabial folds decreased, upper lip posture and support improved, the relationship between the middle and lower facial thirds became more harmonious, and occlusion, nasal breathing, and passive lip seal improved in both patients. Extraoral photographs at rest and while smiling, in frontal and profile views, obtained at 18 months in Case A and at 24 months in Case B, documented the augmentation of the paranasal regions and the maintenance of the aesthetic outcome (Figures 9 and 10).

**Figure 9:**
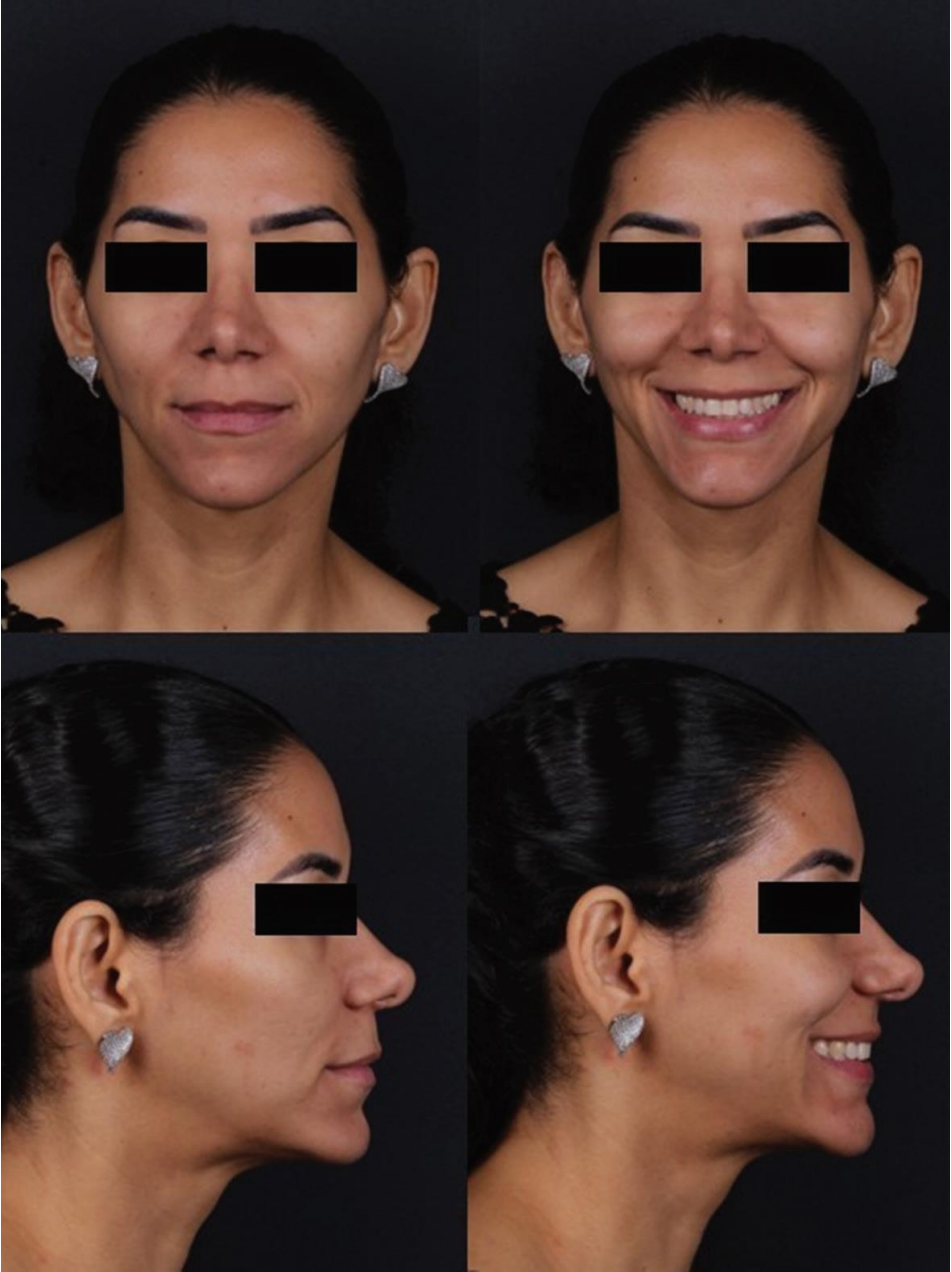
Postoperative extraoral photographs at 18 months—Case A.

**Figure 10:**
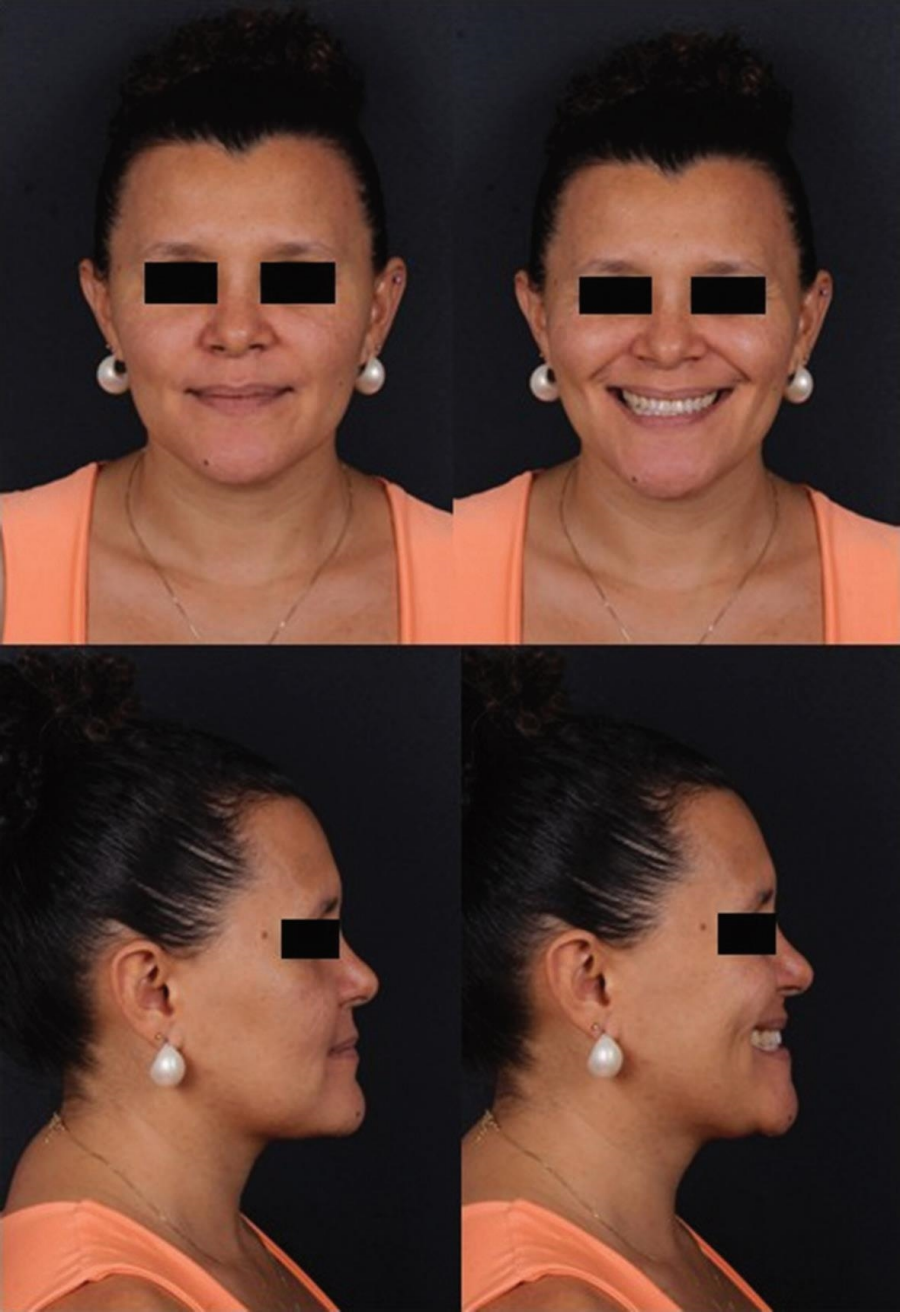
Postoperative extraoral photographs at 24 months—Case B.

### 2.8. Patient Perspective

At the final follow‐up appointments, both patients were invited to describe, in their own words, their perception of the treatment. The patient in Case A reported that her face looked “more balanced and rejuvenated,” that she could close her lips without effort, and that her breathing and sleep quality had improved; she stated that she would undergo the procedure again. The patient in Case B reported satisfaction with the softening of the nasolabial folds and with her dental occlusion, remarking that her profile no longer conveyed the “tired, aged” appearance that had motivated her to seek treatment. Neither patient reported discomfort attributable to the grafted region during the postoperative period.

## 3. Discussion

Repositioning of the maxilla through the Le Fort I osteotomy transfers only part of the skeletal movement to the overlying soft tissues—on the order of 60%—so that the paranasal region may remain deficient and below the patient′s aesthetic expectations even after a well‐executed skeletal correction [29]. In these circumstances, adjunctive procedures may be employed to increase paranasal volume and refine the facial contour [30, 31].

The indication for the adjunctive graft differed between the two cases and deserves comment. In Case A, the maxilla was impacted with virtually no advancement so that no gain in paranasal projection could be expected from the skeletal movement itself; in Case B, the planned advancement of 3.41 mm was judged insufficient to compensate for the depth of the pre‐existing paranasal deficiency. It must be emphasized, however, that both patients underwent complete bimaxillary correction and that, in the absence of a comparison group treated by orthognathic surgery alone, the improvement observed in the nasolabial folds cannot be attributed exclusively to the Sticky Bone augmentation: The relative contribution of the skeletal repositioning and of the graft cannot be isolated with the present design. This attribution limit is inherent to the case report format and is revisited among the limitations below.

Among the available adjunctive techniques, autogenous block grafting is associated with donor site morbidity and the need for fixation hardware [16, 17]. Loose granular biomaterials—bovine xenograft or hydroxylapatite granules applied without a binding vehicle—may migrate into the maxillary sinus owing to unstable adaptation, predisposing to postoperative infection [32]. In contrast, agglutination of the xenograft with PRF‐derived fibrin to form Sticky Bone dispenses with a donor site and stabilizes the material at the recipient bed, resisting displacement into the sinus and soft tissue invagination through the cohesive fibrin network. The platelets and leukocytes entrapped in this network release growth factors that accelerate bone regeneration, exert antimicrobial activity, and support cytokine production and cell migration, reducing the risk of surgical site infection [33, 34].

Custom‐made polyetheretherketone (PEEK) prostheses produced by three‐dimensional printing, porous polyethylene implants, and polymethylmethacrylate implants have likewise been described for paranasal augmentation [10, 35, 36]. These alloplastic options, however, entail risks of encapsulation when adaptation to the bone surface is imperfect, delayed foreign body reactions, uncertain long‐term behavior, and little or no possibility of intraoperative reshaping [18]. Sticky Bone circumvents these drawbacks: Its gelatinous, moldable consistency allows incremental application and close adaptation to bone defects of varied morphology [37, 38]. In Case A, progressive accommodation of the graft was observed over follow‐up, with increasingly intimate adaptation of the biomaterial to the paranasal surface (Figure 7).

Injectable hyaluronic acid [19] and autologous fat transfer [15] show volumetric unpredictability over time because of resorption, frequently requiring repeated interventions. Moussa et al. evaluated horizontal bone dimension and density after Sticky Bone grafting of the anterior maxilla and reported a mean horizontal gain of approximately 2.86 mm at 3 months, with progressive densification up to 6 months [39]. Consistent with those findings, the present cases demonstrated satisfactory dimensional stability of the paranasal graft on tomographic superimposition. In Case A, thickness changed from 6.6 mm (right) and 5.9 mm (left) in the immediate postoperative period to 6.5 and 5.1 mm at 18 months, corresponding to maintenance of 98.5% and 86.4% of the initial thickness. In Case B, the values changed from 8.2 and 7.7 mm to 7.8 and 7.1 mm at 12 months—maintenance of 95.1% and 92.2%—and remained unchanged at 24 months (Figures 7 and 8).

The temporal pattern observed in both patients is consistent with the expected biological behavior of deproteinized bovine bone mineral, whose particle remodeling phase is most active during the first months after grafting and progressively gives way to incorporation and stability [40]. In Case A, the thickness measured at 6 months was slightly greater than that of the immediate postoperative period (6.8 and 6.1 mm vs. 6.6 and 5.9 mm). As this is a particulate graft, such a finding is consistent with the process of incorporation of the biomaterial: During the initial months, the particles agglutinated by the fibrin network undergo remodeling, are progressively surrounded by newly formed bone, and integrate with the recipient bone bed, resulting in closer and more homogeneous adaptation of the graft to the maxillary surface and in a mass of more clearly defined contour on the tomographic sections [24, 40]. At 18 months, thickness was 6.5 and 5.1 mm, corresponding to a minimal net variation relative to the immediate scan. In Case B, all of the measured variation was concentrated within the first year, with no change whatsoever between the 12‐ and 24‐month examinations—a finding that reinforces graft consolidation and long‐term dimensional stability. Minor asymmetries between sides and between patients may be attributed to two local factors: The amount of graft inserted was estimated clinically by visual assessment and may have differed slightly between sites, and the extent of periosteal detachment required by each osteotomy design imposes distinct local vascular demands, being greater in Case B, which underwent a three‐segment Le Fort I osteotomy. In neither case was there any systemic or infectious complication, and the measured variations were of small magnitude, with no perceptible clinical repercussion on paranasal projection or on the patients′ satisfaction, whose aesthetic outcomes remained stable at 18 and 24 months (Figures 9 and 10).

Isolated regional osteotomies have also been proposed to enhance the paranasal contour, but the resulting bone movements exert limited influence on the soft tissues [4, 5]. Furthermore, osteotomy modifications designed to increase paranasal projection [2, 3] require wider surgical exposure, which conflicts with minimally invasive philosophies of orthognathic surgery. Sticky Bone, in contrast, can be introduced and adapted even through restricted surgical access, irrespective of the osteotomy design employed.

The choice of deproteinized bovine bone mineral as the particulate component also warrants explicit justification. Autogenous bone remains the biological gold standard because it is osteogenic, osteoinductive, and osteoconductive; nevertheless, it requires a donor site with additional morbidity and operative time, provides a limited volume, and may undergo unpredictable resorption when used as an onlay graft [16, 17]. Synthetic substitutes eliminate donor site morbidity but display variable osteoconductivity, handling properties, and long‐term volumetric behavior, and loose alloplastic granules have historically been associated with displacement and infection in the paranasal region [18, 32]. Deproteinized bovine bone mineral, in turn, is widely available at comparatively low cost, is biocompatible and osteoconductive, and is characterized by slow resorption [40]—a property of particular value in an aesthetic indication that depends on long‐term volumetric stability. Its granular presentation permits homogeneous agglutination with liquid fibrin within the moldable Sticky Bone matrix, and the combination with PRF compensates for the lack of osteoinductive potential of the xenograft by adding autologous growth factors, leukocytes, and a fibrin scaffold that favors angiogenesis and cell migration [22, 41]. This combination of biological, practical, and economic attributes motivated our preference over autologous harvesting and synthetic substitutes.

The regenerative applications of Sticky Bone are well documented, particularly in implant dentistry [42, 43], including maxillary sinus floor elevation [41, 44, 45] and horizontal and vertical ridge augmentation by guided bone regeneration. The biomaterial releases substantial amounts of cytokines and growth factors that participate in healing and tissue recovery, and it offers the practical advantages of easy handling and reduced operative time [43]. These properties support its use for paranasal volume enhancement in the cases reported here.

The L‐PRF membranes contributed to the stability of the construct by providing an additional barrier against displacement of particles into the maxillary sinus. Such membranes actively release high concentrations of growth factors and cytokines for up to 28 days after preparation, through platelet activation and the consequent secretion of vascular endothelial growth factor (VEGF), transforming growth factors *β*1 and *β*2 (TGF‐*β*1 and TGF‐*β*2), bone morphogenetic proteins (BMPs), insulin‐like growth factor (IGF), and platelet‐derived growth factor (PDGF) [41, 44–46].

The resorbable collagen membrane positioned over the Sticky Bone assisted in maintaining graft volume and in excluding soft tissue from the grafted site [47]. Its dense layer faces the soft tissues and acts as a cellular barrier, whereas the porous layer faces the graft and helps to stabilize the clot and the particles [48]. Unlike nonresorbable barriers such as PTFE membranes and titanium meshes—which require secondary removal and show higher rates of exposure and infection [49]—collagen membranes require no fixation and are fully resorbed within 4–6 months [47], eliminating any additional procedure.

The technique showed a low potential for complications in the present cases; nonetheless, displacement of graft particles may occur when PRF preparation is inadequate, and bovine‐derived particles are expected to undergo slow resorption over the long term [40]. We consider that, should late resorption of the mineral phase occur, replacement by organized fibrous tissue would tend to preserve the achieved projection; even so, this expectation requires confirmation in studies with extended follow‐up.

The limitations of this report are inherent to its design: It is the initial description of a technique, presented as a case report and, therefore, a preliminary study. The purpose of this report was precisely to describe the technique in a detailed and reproducible manner and to document its clinical and radiographic behavior over a minimum follow‐up period of 1 year, thereby providing the basis for subsequent investigations. Accordingly, the authors are already conducting studies with a larger number of patients and standardized clinical and radiographic follow‐up, which will allow comparative assessment of the additional benefit, long‐term volumetric stability, and patient perception of the technique, and whose results will be the subject of future publications.

## 4. Conclusion

The cases reported here indicate that the Sticky Bone technique is a feasible and effective clinical option for augmenting the paranasal region when the skeletal movements of the Le Fort I osteotomy alone would not deliver the desired aesthetic refinement. The material behaved as a biocompatible, dimensionally stable, and low‐cost graft, while additionally favoring new bone formation between the osteotomized segments. Further studies with longer follow‐up periods and a larger number of patients are needed.

## Author Contributions

**Hugo Leonardo Mendes Barros:** conceptualization, investigation, writing – original draft. **Isabella Mousinho Marinho dos Santos:** investigation, data curation, writing – original draft. **Ana Luisa Rios Barbosa de Almeida Xavier:** investigation, visualization, writing – original draft. **Adelmar Pereira da Silva Neto:** investigation, resources, writing – review and editing. **Thais Pimental de Sá Bahia:** methodology, validation, writing – review and editing. **Daniel Amaral Alves Marliére:** conceptualization, methodology, supervision, project administration, writing – review and editing.

## Funding

No funding was received for this manuscript.

## Disclosure

All authors have read and approved the final version of the manuscript. Daniel Amaral Alves Marliére, the corresponding author, had full access to all of the data in this study and takes complete responsibility for the integrity of the data and the accuracy of the data analysis.

## Ethics Statement

This article is a retrospective report of two cases treated according to standard clinical care; no experimental intervention or protocol deviation was involved. In accordance with institutional policy, formal research ethics committee approval is not required for fully anonymized retrospective case reports of routine care accompanied by the patients′ written informed consent, and it was, therefore, not sought. All procedures were conducted in accordance with the ethical standards of the institution and with the 1964 Declaration of Helsinki of the World Medical Association and its later amendments.

## Consent

Written informed consent was obtained from both patients for the surgical treatment and for the publication of this report and the accompanying images. The signed informed consent forms are being submitted to the journal together with this manuscript.

## Conflicts of Interest

The authors declare no conflicts of interest.

## Data Availability

The data that support the findings of this study are available from the corresponding author upon reasonable request. The data are not publicly available owing to privacy and ethical restrictions involving identifiable patient information.
